# Patients’ Experiences of Care With or Without the Support of an Interactive App During Neoadjuvant Chemotherapy for Breast Cancer: Interview Study

**DOI:** 10.2196/39983

**Published:** 2022-08-11

**Authors:** Maria Fjell, Ann Langius-Eklöf, Marie Nilsson, Kay Sundberg

**Affiliations:** 1 Department of Neurobiology, Care Sciences and Society Karolinska Institutet Stockholm Sweden

**Keywords:** breast cancer, neoadjuvant chemotherapy, experiences of care, mobile health, mHealth, mobile app, patient participation, mobile phone

## Abstract

**Background:**

Neoadjuvant chemotherapy (NACT) is often recommended for patients with breast cancer with more aggressive tumor characteristics. As with all chemotherapies, they can cause substantially disturbing symptoms. Most patients receive their treatment as outpatients, which means that they must take responsibility for self-care and management of symptoms at home for a long period. Patients with breast cancer undergoing chemotherapy may not receive sufficient support for management of treatment-related symptoms. For patients undergoing NACT, it has been concluded that information and supportive needs are not always met. In our previous study, the use of mobile health to support patients with breast cancer undergoing NACT reduced symptoms during treatment with the support of an interactive app. Therefore, it is important to investigate how patients experience their care and explore any specific contribution that the app may have brought in care.

**Objective:**

This study aims to explore patients’ experiences of care with or without the support of an interactive app during NACT for breast cancer.

**Methods:**

This qualitative study was part of a larger randomized controlled trial and included 40 individual face-to-face interviews conducted with patients in both intervention and control groups after the end of NACT. The interviews were audio recorded, and the data were analyzed inductively using thematic analysis.

**Results:**

No major differences in experience of care were observed between the groups. A total of 4 overarching themes emerged. In the first theme, *The health care context*, patients described care as assessible, although sometimes there was a lack of time and continuity with nurses. In the second theme, *Being a recipient of care*, it emerged that the patients experienced a warm and positive atmosphere at the clinics. In the third theme, *Taking an active role as a patient*, patients described being active in searching for information and various ways of participation in their own care. In the fourth theme, *The value of the app*, patients who had used the app experienced it as a complementary source of information, creating a sense of security. Using the app provided patients with the support of being contacted by a nurse if needed, enabled self-care, and facilitated the planning of daily activities.

**Conclusions:**

Overall, patients’ experiences of care were similar and mostly positive. However, for patients using the app, it provided additional support for information and self-care and enhanced participation in their own care. The easy access to a nurse gave patients a sense of security. The findings suggest integrating an interactive app as a complement to standard care to support patients with breast cancer during treatment.

**International Registered Report Identifier (IRRID):**

RR2-DOI: 10.1186/s12885-017-3450-y

## Introduction

Patients with breast cancer with more aggressive tumor characteristics are often treated using neoadjuvant chemotherapy (NACT), which is administered before surgery [1,2]. The main purpose of NACT is to reduce the tumor size, known as downstaging. This may allow less extensive surgery on the breast and axilla, thereby facilitating breast-conserving surgery instead of mastectomy, as well as improve cosmetic outcomes and reduce postoperative complications such as lymphedema [3,4]. NACT also allows early treatment of possible micrometastases and provides valuable prognostic information regarding the effectiveness of treatment [1,5]. The treatment is considered both safe and effective [1,6-8]. However, as with all chemotherapies, it can cause substantial physical and psychological symptoms [9,10].

In general, most patients with breast cancer receive treatment as outpatients, which means self-care and management of symptoms at home for a long period [11,12]. This means that patients are expected to participate in their own care, and this involves patient learning to obtain knowledge and skills to manage illness and symptoms in collaboration with the nurse [13]. It also includes a caring relationship between the nurse and patient built on reciprocity and trust [14]. However, it is evident that patients have different needs concerning participation [15]. There are patients who are satisfied with not being so active but also those who express that they want to participate in their own care, and for achieving that, there are both facilitators and barriers [16,17]. Participation should be considered on an individual basis, according to the patient’s specific situation [18].

In contexts with short hospital stays and outpatient treatments, patients need to actively engage in self-care, but they need support in managing this condition [13,19]. Previous studies have shown that patients with breast cancer may not receive sufficient supportive care for treatment-related symptoms during chemotherapy [20,21]. Studies regarding patients’ needs during NACT are few, and they report that information and supportive needs are not always met [22,23]. This may result in impaired well-being, reduced health-related quality of life, distressing visits to emergency departments, hospitalizations, and poor treatment outcomes [22,24]. Therefore, ensuring that the care needs are identified, assessed, and managed is imperative.

Technical advances in the field of mobile apps and web-based systems have led to an increased use of mobile health (mHealth) to improve the delivery of health care and to support patients with cancer [25,26]. Studies on the use of such technology have shown decreased symptom burden, improved health-related quality of life, and increased survival [26,27]. We developed an interactive app (Interaktor) for smartphones and tablets, with the intention of supporting patients in real time during cancer treatment regarding symptom management [28]. The patients showed high adherence and engagement in using the app, which promoted continuous contact with the nurse [29] and led to less symptom burden during treatment of prostate and pancreatic cancer [30,31]. In a randomized controlled study using the Interaktor app during NACT, the results showed lower symptom prevalence and symptom distress and better emotional functioning than the control group 2 weeks after the end of treatment [32]. The next step in evaluating the use of the app was to explore whether the app contributed to standard care in any specific way. This study aimed to explore patients’ experiences of care with or without the support of an interactive app during NACT for breast cancer.

## Methods

### Study Design

In this study, a qualitative design was applied to explore patients’ experiences of care and the significance of using the app. This study is part of a larger randomized controlled trial (RCT; ClinicalTrials.gov NCT02479607) evaluating the Interaktor app in patients undergoing NACT for breast cancer [28].

### Sample and Setting

In the larger RCT, 149 patients diagnosed with breast cancer and treated with NACT were included in an intervention group (n=74, 49.7%), using the app Interaktor in combination with standard care, or a control group (n=75, 50.3%), only receiving standard care [32]. Inclusion criteria were as follows: aged ≥18 years, diagnosed with nonmetastatic breast cancer planned for NACT, able to read and understand Swedish, and no medical condition of cognitive dysfunction. The trial was conducted at 2 university hospital oncology clinics in Stockholm, Sweden.

When agreeing to participate in the RCT, the patients were informed by the researcher that they could later be contacted and invited to participate in an interview study about their experiences of care during NACT and the significance of the app among patients who had used it during the study. Three months after the end of NACT, a consecutive sampling strategy for the interviews was adopted in the first 20 patients, with an equal number of patients from both the intervention and control groups at the 2 hospitals. Subsequently, a strategic sampling strategy was used to capture a range of patient characteristics based on group, age, marital status, educational level, occupation, and treatment duration in weeks. A final sample of 40 patients from the intervention (n=21, 53%) and control (n=19, 47%) groups was included in this study (Table 1). There were no statistically significant differences in the sociodemographic and clinical characteristics at baseline between the 2 groups.

**Table 1:** Sociodemographic and clinical characteristics at baseline (N=40).

Characteristic		Intervention group (n=21)	Control group (n=19)
Age (years) at inclusion, mean (SD; range)		51.7 (12.5; 30-73)	54.2 (13.5; 35-77)
**Marital status, n (%)**			
	Married or cohabiting	17 (81)	14 (74)
	Living alone	4 (19)	5 (26)
**Education level, n (%)**			
	University	13 (62)	11 (58)
	Secondary school	3 (14)	6 (32)
	Primary school	5 (24)	2 (10)
**Occupation, n (%)**			
	Working	16 (76)	13 (68)
	On sick leave	2 (10)	1 (5)
	Retired or unemployed	3 (14)	5 (26)
NACT^a^ duration in weeks, mean (SD; range)		15.3 (1.9; 11-20)	15.6 (2.5; 11-23)

^a^NACT: neoadjuvant chemotherapy.

### Standard Care

Standard care consists of treatment and care according to national care guidelines, including visits to the physician at the oncology clinic before each chemotherapy treatment, approximately every second or third week, depending on the chemotherapy regimen. Moreover, the patient is assigned a contact nurse who has the overall responsibility for the patient throughout the care chain. The contact nurse provides the patient with information about the treatment and the planning of care during a scheduled visit before the start of treatment. During treatment, the contact nurse supports patients with information, establishes a care plan, assesses the patient’s symptoms and needs, and takes actions based on these symptoms and needs. In case of questions or concerns related to treatment, the contact nurse is available during office hours. During other hours, patients are referred to the oncology emergency unit or inpatient or emergency department, depending on which hospital the patient is being treated at [33].

### The Intervention With Interaktor for Patients With Breast Cancer During NACT

The content of Interaktor for NACT was developed through literature reviews, clinical guidelines, and discussions and consultations with health care professionals [32]. The app, running on a smartphone or tablet, has several features: self-reporting of 14 commonly prevalent symptoms during chemotherapy, the transfer of the reported symptoms to a secure server, a web interface where a nurse can monitor the patient’s reports in real time, a risk assessment model for symptoms of concern that sends alerts to a nurse at the clinic by an SMS text message, and continuous access to evidence-based self-care advice and relevant websites related to assessed symptoms and other areas of concern. Moreover, the patients could monitor their own reported symptom history over time in graphs. When alerted, the nurse calls the patient to discuss the symptoms and their management. If an alert is triggered, a notification suggests that the patient reads the related self-care advice [34]. During the RCT, the patients reported symptoms daily on weekdays (8 AM-4 PM), starting on their first day of NACT and continuing until 2 weeks after the end of NACT, approximately 18 weeks in total. More details and illustrations of the app have previously been presented [28].

### Data Collection

Data collection for this study was conducted between January 2016 and August 2017. The interviews were conducted by the first and third authors (MF and MN) and an additional researcher. All interviews took place in a secluded room at the 2 oncology clinics 3 months after the end of NACT. A semistructured interview guide was used, covering different aspects of patient participation such as the relationship between patients and nurses, patients’ information needs, self-care, and caretaking [18,35]. In addition, the patients from the intervention group were asked about the significance of using the app during treatment. The patients were asked to speak as freely as possible around each question, and depending on the extent of the answers, follow-up questions were used (Textbox 1). The interviews were audio recorded and lasted between 14 and 61 minutes, with a median duration of 27 minutes.

**Interview guide.**

**Question and follow-up questions:**

1. How did you experience the contact (care relationship) between you and the nurse during the treatment period?

- How has it been? Give examples.
- Has it come about naturally or have you and/or your relatives been forced to bear the weight of your care? Give examples.
- Has the nurse considered your experiences/wishes about your care? Give examples.
- Did you get help when you needed it? Give examples.

2. Do you feel that you have received enough information regarding your care and treatment? Give examples.

- How has the information been provided (written, verbal, over the phone, or during visits)?
- When was the information given?
- Was the information provided in such a way that you understood and could absorb it? Give examples.
- Did you lack any information? Give examples.

3. How did you experience your encounters with the nurse?

- Were you given enough time with the nurse?
- Did you feel you were taken seriously/respected? Give examples.

4. Have you been involved in your care?

- Can you describe how you have been involved or not involved?

5. Was there a dialogue in your meetings with the nurse? Give examples.

- In what way have you had the opportunity to express how you wanted your care/treatment to be?
- Have you had the chance to ask questions or express concerns? Give examples.
- Has the nurse considered any of your experiences/wishes in the planning of your care? Give examples.

6. Have you received advice and help on how to treat symptoms or other concerns? Give examples.

- Did the nurse explain the cause of the symptoms? Give examples.
- Did the nurse explain how the symptoms should be managed? Give examples.
- How did you experience the information given by the nurse?
- Did the advice help? Give examples.
- Did you get help with other basic needs (eg, sick leave and counseling)? Give examples.

7. Is there anything you would like to change in health care? Give examples.

8. What significance did the app have for you during the treatment? (Note: this question concerns the intervention group.)

- What significance did the app have for your involvement in care?

9. Is there something you would like to add before we finish the interview? Give examples.

### Ethics Approval

This study was approved by the regional ethical review board of Stockholm, Sweden (registration numbers 2013/1652-31/2 and 201712519-32).

### Data Analysis

**Figure 1:**
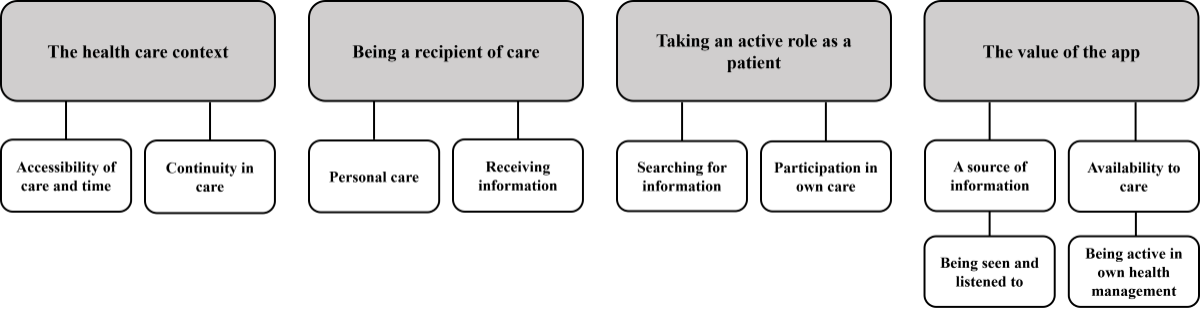
Overarching themes (gray rectangles) and subthemes (white rectangles) identified through the thematic analysis of interviews with the patients (N=40).

The interviews were analyzed with an inductive approach using thematic analysis described by Braun and Clarke [35,36]. The recorded interviews were transcribed verbatim, and the texts were read several times to become familiar with the data as a whole. Each group (intervention and control) was analyzed separately by the first and second authors (MF and ALE). Statements from the patients in agreement with the study objective were systematically coded throughout the entire data set of each patient and transferred into a coding sheet. A code consisted of a few words or whole sentences. The codes from each group were then discussed by the 2 authors. As there were few differences in the codes concerning experiences of care between the 2 groups, the codes were merged into one coding sheet and tagged with an identification so that they could be distinguished. The analysis was continued by sorting the matching codes from both groups into areas. The areas were reviewed so that they covered all codes. Subsequently, the areas were analyzed into themes. The themes were then discussed, reviewed, and revised several times to ensure that they worked well in relation to the areas with included codes. Finally, the themes were defined, named, and renamed, resulting in 4 overarching themes and 10 subthemes (Figure 1). Throughout the entire analytic process and during the writing of the manuscript, all authors (MF, ALE, MN, and KS) continuously discussed the analysis to increase trustworthiness. To illustrate the findings, examples of individual quotes from patients are presented in the Results section.

## Results

### Overview

Regardless of whether the patients had used the app, few differences emerged in the patients’ experiences of care within the themes, and both groups reported both positive and negative experiences. The descriptions of patients’ experiences of care are presented in three overarching themes: *The health care context*, *Being a recipient of care* and *Taking an active role as a patient.* The significance of the app for patients is described in the overarching theme *The value of the app*.

### The Health Care Context

#### Accessibility of Care and Time

Patients who had experienced accessibility to care knew who they should contact when needed, and they stated that it had been easy to get in touch with the nurse. A patient stated the following:

I had all the contact information I needed. If there was something acute or if I needed contact during the weekend, I had information on where to call and reached the right department instantly. So, it has really been a comfort.

Patient 53, Intervention group

The patients said that they had received sufficient time from the nurse during visits or over the telephone. Patients who had experienced accessibility difficulties described that they had not received specific contact information, and it was difficult to get in touch with the nurse owing to staff shortages. Sometimes, their calls were returned several days later or not at all, leading to frustration. This was described as follows:

It was hard not being able to get in direct contact with the contact nurse...I was frustrated having to wait so long to be called.

Patient 5, Control group

Occasionally, patients did not get enough time to consult with the nurse or the opportunity to ask questions during visits or over the phone.

#### Continuity in Care

Having a contact nurse was valuable for good continuity and was a great support during the treatment period, as exemplified by the following quote:

Having the same contact nurse was a comfort that meant a lot...everything became easier when I met familiar faces.

Patient 6, Control group

Lack of continuity was described as having to meet too many different nurses and physicians or not knowing who their contact nurse was, which in turn led to confusion and feelings of insecurity regarding whether the nurse was in control or not. A patient described this as follows:

It was a bit confusing because I had a change of contact nurse four times and I have had four different physicians. There has been no continuity if you know what I mean.

Patient 58, Intervention group

### Being a Recipient of Care

#### Personal Care

The atmosphere at the oncology clinics was perceived as friendly, positive, confirming, and warm, which was not commonly experienced elsewhere. The patients could laugh and have fun with the nurse even though they were receiving treatment for cancer. A good dialogue with the nurse where both parties could ask and answer questions as well as having discussions even if they had different opinions were considered crucial. The patients described being listened to and treated with respect and empathy by the nurse, which led to trust, safety, and encouragement to keep up with treatments. The following quote describes how a patient experienced it:

They told me that we will make sure this becomes a parenthesis in your life. And exactly those words I took note of, which made it feel like there was a positive future in some way.

Patient 15, Intervention group

Negative experiences such as a sense of coldness and not being taken seriously were also described:

I felt a sense of coldness in the beginning when I needed a hug instead.

Patient 44, Control group

Experiencing a lack of dialogue about symptoms or concerns or having to remind the nurse repeatedly regarding, for example, booking appointments or referrals to counseling or prescriptions of medicines, gave feelings of being only one in the crowd.

#### Receiving Information

The patients were generally satisfied with the verbal and written information they had received as well as answers to questions about the treatment and related symptoms, self-care, and future planning. This created a notion of being prepared and knowing what to expect during treatment:

I never felt anxious or nervous because I knew what would happen and how I might feel.

Patient 3, Control group

Sometimes, there was a lot of information, which was hard to take in and keep track of. In contrast, some considered the information to be insufficient in certain areas or felt that they had to nag for answers to their questions. On a few occasions, patients felt that information was withheld regarding why NACT was chosen for them specifically instead of surgery and how the treatment affected the whole body. As a patient stated the following:

I do not think the whole picture of my disease was explained to me. I had the feeling of being withheld information...I wanted to know everything, so I asked for my medical records to try to understand.

Patient 30, Intervention group

### Taking an Active Role as a Patient

#### Searching for Information

Some patients described that they actively searched for more information than what was provided by the nurse. Usually, the internet was used to search for information about the illness, treatment, and other patients’ experiences of the treatment to reassure themselves as to whether symptoms and signs were normal or not:

I found out a lot of different things myself about what was going to happen, why I felt like I did and so on. I care about my own body.

Patient 1, Intervention group

The pharmacy was also a source of more information regarding prescribed medications. Moreover, patient organizations focusing on patients with breast cancer were used, especially to get in contact with and receive information from persons who have had breast cancer and have undergone treatment.

#### Participation in Own Care

The patients described their participation in care in various ways. Not being an active participant was commonly mentioned, albeit positively; they had accepted the situation and had no need to influence, choose, or have specific requests about their care. The highest priority was to get well from a serious disease, and they had accepted the plan and trusted the nurse to recommend what was best for them:

I accepted and followed what they recommended for me and that worked. I had no need to influence my care and treatment.

Patient 23, Control group

Sometimes, treatment decisions and future planning were presented as a package at an early stage, and patients felt that their participation was not requested. A patient stated the following:

You were told that you should participate in the care. I did not know what options I had, or I could say that no alternatives were presented for me. The treatment and the planning were already decided and presented for me, and I accepted it.

Patient 33, Intervention group

There were patients who described participation as following advice from the nurse. Realizing that they could do something themselves helped them feel better and made the treatment more manageable. Furthermore, having a treatment plan facilitated their daily planning. Having the possibility of discussing different matters regarding their care was also important for their feelings of involvement.

### The Value of the App

#### A Source of Information

The patients described using Interaktor as an easy and accessible source of information where symptoms caused by the treatment were explained. Patients could follow the related self-care advice instead of contacting the contact nurse for information about how to manage their symptoms. A patient said the following:

Using the app led me to get information, for example about the mouth, which I had huge problems with. I could read about common symptoms and then there were recommendations on what I could eat and do myself. I thought it also helped me to seek even more information.

Patient 15, Intervention group

Thus, the information in the app was a good complement to the verbal and written information provided by the nurse during treatment and created a sense of security. The links to the websites in the app felt safe and useful for obtaining more in-depth information.

#### Availability of Care

Using Interaktor was experienced by the patients as an easy and straightforward way to reach the contact nurse, as they were contacted directly in the event of severe symptoms or concerns. This was described as safe because they knew that they would be contacted by the nurse when they felt ill and because they experienced that they were contacted quickly:

Well, the times when I needed help, I got it right away. Otherwise, without the app, I had to call, and they called me back. With the app, they basically called ten minutes later.

Patient 25, Intervention group

Furthermore, being contacted in the event of an alert was much more convenient than having to look for the right telephone number for the contact nurse.

#### Being Seen and Listened To

Reporting symptoms in the app facilitated the patients to share information about their health condition with the nurse, who could then monitor the patients’ reports. The patients also stated that in the event of severe symptoms that led to contact with the nurse, they had the opportunity to ask questions and discuss any problems. Thus, the patients felt that they were being monitored in a positive way and led to feelings of being seen and listened to and not being on their own:

For me, it contributed with feelings of not being cast aside or of being alone. There was someone who actually saw what I reported and provided feedback. For me, this gave me a sense security I would say, a stability.

Patient 6, Intervention group

#### Being Active in Own Health Management

The patients described being active by reporting symptoms daily, as opposed to contacting the nurse themselves when they felt unwell. Furthermore, the app facilitated self-care actions when symptoms occurred, to increase well-being. Reporting and monitoring symptoms in the graphs provided patients with an opportunity to reflect on how they felt every day. Being more conscious and aware of their symptoms enabled them to see patterns and fluctuations in their symptoms. This also facilitated their planning of activities and created positive feelings and comfort when they discovered that many days were trouble free. Reporting and keeping track of symptoms in the app was described as a way of keeping a diary:

For me, it was good to be able to go back and check how many days I felt ill, and when I came back to the clinic, I could say that I had felt really ill two weeks after the treatment, and then it became a bit better. So, for me, given that my memory does not work, I think that was supportive.

Patient 30, Intervention group

## Discussion

### Principal Findings

To the best of our knowledge, this is the first study to specifically explore experiences of care in patients with breast cancer during NACT and investigate the impact of an interactive app on standard care. Overall, patients from both groups expressed positive experiences with their care during NACT, although negative experiences were also mentioned. The patients in the intervention group experienced that using the app provided added value to standard care with regard to additional support for information, self-care, and enhanced participation in their own care. In Sweden, patients who receive NACT are treated according to national care guidelines, and the role of the contact nurse is to support patients throughout the chain of care [33]. However, results of this study are in congruence with those of other studies showing that patients request more support during their treatment in addition to what the health care offers [37]. Commonly, this support concerns information and how to manage symptoms caused by the illness and treatment [38]. Presently, the patients described the Interaktor app as providing extra support during the treatment period by having easy access to self-care advice, information, and contact with the nurse if the symptoms were severe. The results are in line with those of the recent reviews where patients with cancer experienced apps as supportive tools that complemented or extended existing health care [39,40].

Although most of the patients felt well informed, a lot of information was given at the same time, which sometimes was hard to absorb. Studies have shown that patients undergoing treatment for cancer have a great need for relevant information to gain an understanding of the treatment [41-43]. Patients receiving chemotherapy often have trouble concentrating and difficulties remembering [44]. Supportive mHealth apps can thus be convenient for patients during treatment, as they can retrieve information to refresh their memory whenever needed.

We have previously reported increased symptom relief when using Interaktor [32], and the results of this study testify that the app was a facilitator for patients to participate in their own care. By reporting and monitoring their symptoms as well as using easily accessible self-care advice, they had been active in relieving their symptoms. Another benefit of using the app, mentioned by some, was the graphs showing the course of their symptoms, which was useful in planning everyday life. Furthermore, accessibility to nurses during treatment was essential for patients, especially in the case of severe symptoms. Accessibility to care characterizes how easily a patient can reach the health care provider. Availability refers to the extent to which the health care provider has the resources to be reached; for example, through personnel and technology [45]. Presently, Interaktor has served as a safe and convenient tool for achieving both.

Most patients described their dialogue with the nurse as respectful, encouraging, and personal. Using Interaktor reinforced patients’ experiences of being seen, listened to, and feeling safe. Similarly, improved patient safety and increased communication between patients and health care providers were shown in a recent review of patients with cancer using mHealth [46]. Results from other studies on cancer care have shown that an established good relationship between the patient and nurse is vital for the patient to feel acknowledged, which also facilitates patient participation [18,44]. Interestingly, in this study, it was common for patients to be satisfied with their own care without the need to influence or have special requests for care. In contrast, some appreciated being active in searching for information and engaging in self-care. This indicates that patients may not be aware that there are many ways for them to participate in their own care. Studies have confirmed that the meaning of concept of participation is vague and needs to be clarified to be practically achieved in a clinical context [14,47].

### Strengths and Limitations

The selected sample was considered to be ample in size and heterogeneity to provide richly textured information for trustworthiness of data. A thematic analysis method was chosen, providing a structured approach to handle the large data sets and to identify patterns across the data set [48,49]. In addition, by conducting most of the interviews and transcriptions, the first author, who also played a major part in the analysis, became acquainted with the material. The risk of the first author’s preunderstanding was considered by confirming the transparency of the analysis with the other authors. The fact that the interviews were conducted 3 months after the end of NACT could have caused recall bias; however, the richness of the data testifies against this bias.

### Conclusions

In this study, it was evident that patients felt well taken care of and mostly had positive experiences of their care during their treatment for breast cancer. This was regardless of whether they had used the app. The results show that there is potential for improvements in how information, communication, and access to a nurse are delivered in care. Patients using the interactive app experienced this as an added value during their treatment. The extra support for information and self-care enhanced participation in their own care, and easy access to nurses gave them a sense of security. These findings suggest that there are good reasons to integrate an interactive app as a complement to standard care to support patients treated for breast cancer. Further investigation should be conducted on nurses’ experiences of the intervention with Interaktor and how it impacts their work. Moreover, an evaluation of the cost-effectiveness of the app is warranted.

## Abbreviations

mHealth: mobile health
NACT: neoadjuvant chemotherapy
RCT: randomized controlled trial
